# Patent Crystalline Sponge Gold

**Published:** 1853-07

**Authors:** A. J. Watts


### ARTICLE XV.

### *Patent Crystalline Sponge Gold.*

The following is the specification of Letters Patent, issued to Alfred J. Watts, April 26th, 1853.

*To all whom it may concern.*—Be it known, that I, Alfred J. Watts, of the city of Utica, in the county of Oneida, and state of New York, have invented a certain new and improved mode of preparing and crystallizing gold, and I do hereby declare that the following is a full, clear and exact description thereof.

The nature of my invention consists of dissolving gold, (which has previously been purified by any of the usual and well known methods) in mercury, and after treatment by heat, or otherwise, dissolving out the mercury by nitric acid, and then subjecting the new conditioned, but as yet unfinished gold to the action of a particular heat, whereby it is rendered coherent, soft and malleable, admirably fitting it for the purpose of filling teeth.

To enable others skilled in the art to make and use my invention, I will proceed to describe the process by which the gold is prepared.

I take gold, either pure or alloyed, dissolve it in nitro-muriatic acid as usual, and precipitate by proto-sulphate of iron. I wash the precipitated gold with diluted hydro-chloric acid, to remove any peroxyd of iron, or other impurities, edulcorate with hot water, and dry it thoroughly.

I now amalgamate it with from 4 to 12 times its own weight of mercury, triturate it thoroughly, and then set it one side, and allow it to stand for from one hour to twenty-four hours, according to circumstances.

If I wish the gold to be in a highly crystalline condition, I make a pretty fluid amalgam, and after thorough trituration, put it in a flat bottom vessel, and heat it gradually till it is quite hot and painful to the touch, say from 180° to 240° fahr. I keep it at this heat for a few minutes, and then allowing it to cool gradually, let it remain some hours as before said to condition itself. I then pour over it pure nitric acid, diluted with about its own bulk of water. I apply heat very gently at first, and as the action progresses, I increase it. Towards the end of the operation, when the mercury appears to be all dissolved out, and the gold presents the appearance of a mass of crystals, or semi-crystals, sponge, &c., I pour off the acid solution of mercury, and pour pure undiluted nitric acid into the vessel containing the gold, and apply heat. This dissolves out entirely the mercury, or any other metals which may have escaped the action of the diluted acid, and also any of the salts of mercury remaining in the pores of the gold. And after washing with hot water and drying, the gold is left in perfectly pure condition. But in this present state, it is very friable, non-coherent, and so easily broken down, that it will not bear the slightest handling without breaking up into a fine powder, and must be very tenderly treated while getting in a positon to be subjected to the next process.

When this is thoroughly dry, I raise the heat to a cherry-red, or to a heat just short of the melting point of gold. This is a particular part of the process, and requires care and skill. The heat must be raised just to that point which will partially liquify without actually melting the gold, and when properly managed, the gold will be left in the condition of a soft, malleable and extremely ductile mass of crystals, which will be either close and spongy, or loose and in a mass of brilliant, needle shaped crystals, radiating from centres and crossing each other in every direction, and will bear handling without crumbling to pieces, and upon pressure will readily weld into a solid mass, eminently fitting it for the purpose set forth.

I take gold, either pure or alloyed, (I prefer pure,) roll it out into thin strips, heat them to a red heat, cut them up into small pieces, put them into a glass, a matrass, or any convenient vessel to answer the purpose hereinafter mentioned. I pour over it from 6 to 10 times its weight of mercury, apply heat just short of the boiling point of mercury. The vessel is closed and kept cool at the top, so as to condense any mercurial vapors, and the gold dissolves. I pour it into a glass mortar, and afterwards add more mercury, according to circumstances, and triturate it thoroughly till cold, and pour it into a flat bottomed vessel convenient for applying nitric acid. I then, according to the condition I wish to bring the gold into, either apply heat and set it one side to cool gradually or quickly, as required, or set it one side without applying heat, to remain an hour or a day, according to circumstances, and then apply the acid as in other cases before mentioned.

What I claim as my invention, and desire to secure by Letters Patent, is, the within described processes of preparing, or crystallizing gold, for the purpose of filling teeth, substantially as herein set forth and described.

A. J. WATTS.

J. L. Smith, } Witnesses.

E. G. Dennis, }

Patented, 26th April, 1853. [*N. Y. Dent. Rec.*

